# Population dynamics of *Anopheles gambiae s.l.* in Bobo-Dioulasso city: bionomics, infection rate and susceptibility to insecticides

**DOI:** 10.1186/1756-3305-5-127

**Published:** 2012-06-21

**Authors:** Roch K Dabiré, Moussa Namountougou, Simon P Sawadogo, Lassina B Yaro, Hyacinthe K Toé, Ali Ouari, Louis-Clément Gouagna, Frédéric Simard, Fabrice Chandre, Thierry Baldet, Chris Bass, Abdoulaye Diabaté

**Affiliations:** 1 Institut de Recherche en Sciences de la Santé/Centre Muraz, 01 BP 390, Bobo-Dioulasso 01, Burkina Faso; 2 IRD/UR016-CRVOI, 2 rue Maxime Rivière 97490, Sainte Clotilde, Ile de la Réunion, Montpellier, Cedex 5, France; 3 IRSS/UMR MIVEGEC/IRD, BP 545, Bobo-Dioulasso, Burkina Faso; 4 LIN-IRD/UMR MIVEGEC, BP 64501, 34394, Montpellier, Cedex 5, France; 5 Centre Entomologique de Cotonou/IRD/CIRAD, 06 BP 2604, Cotonou, République du Bénin Cotonou, Bénin; 6 Biological Chemistry and Crop Protection, Rothamsted Research, Harpenden, AL5 2JQ, UK

**Keywords:** Malaria, *Anopheles gambiae s.l*., *An. arabiensis*, Insecticide resistance, Bobo-Dioulasso, Burkina Faso

## Abstract

**Background:**

Historical studies have indicated that *An. gambiae s.s.* is the predominant malaria vector species in Bobo-Dioulasso the second biggest city of Burkina Faso (West Africa). However, over the last decade, *An. arabiensis* appears to be replacing *An. gambiae s.s.* as the most prevalent malaria vector in this urban setting. To investigate this species transition in more detail the present study aims to provide an update on the malaria vector composition in Bobo-Dioulasso, and also the *Plasmodium* infection rates and susceptibility to insecticides of the local *An. gambiae s.l.* population.

**Methods:**

An entomological survey was carried out from May to December 2008 in Dioulassoba and Kodeni (central and peripheral districts respectively), which are representative of the main ecological features of the city. Sampling consisted of the collection of larval stages from water bodies, and adults by monthly indoor residual spraying (IRS) using aerosol insecticides. Insecticide susceptibility tests were performed using the WHO filter paper protocol on adults emerged from larvae. PCR was used to determine vector species and to identify resistance mechanisms (*kdr* and *ace-1*^*R*^). The *Plasmodium* infection rate was estimated by ELISA performed on female mosquitoes collected indoors by IRS.

**Results:**

*An. arabiensis* was found to be the major malaria vector in Bobo-Dioulasso, comprising 50 to 100% of the vector population. The sporozoite infection rate for *An. arabiensis* was higher than *An. gambiae s.s.* at both Dioulassoba and Kodeni. *An. gambiae s.l.* was resistant to DDT and cross-resistant to pyrethroids at the two sites with higher levels of resistance observed in *An. gambiae s.s.* than *An. arabiensis*. Resistance to 0.1% bendiocarb was observed in the *An. gambiae s.s.* S form but not the M form or in *An. arabiensis*. The L1014F *kdr* mutation was detected in the two molecular forms of *An. gambiae s.s.* at varying frequencies (0.45 to 0.92), but was not detected in *An. arabiensis,* suggesting that other mechanisms are involved in DDT resistance in this species. The *ace-1*^*R*^ mutation was only detected in the S molecular form and was observed at the two sites at similar frequency (0.3).

**Conclusions:**

Over the last ten years, *An. arabiensis* has become the major malaria vector in Bobo-Dioulasso city where it was formerly present only at low frequency. However, the ecological determinant that enhances the settlement of this species into urban and peri-urban areas of Bobo-Dioulasso remains to be clarified. The impact of the changing *An. gambiae s.l.* population in this region for vector control including resistance management strategies is discussed.

## Background

*Anopheles gambiae* s.s. Giles and *Anopheles arabiensis* Patton are two of the most important malaria vector species in Africa. The two species are members of the *An. gambiae* species complex and are sympatric in many regions of the sub-Saharan tropical savannah [1]. Studies of the spatial distribution patterns of *An. gambiae s.l.* have shown that *An. arabiensis* is distributed across East to West Africa [2,3] occurring in sympatry with the *An. gambiae* S form in East Africa (where the M form is absent) and with both *An. gambiae* S and M forms in West Africa. In West Africa, although the two forms of *An. gambiae* are found in sympatry with *An. arabiensis* they exploit different ecological niches [4,5]. The M form is found occupying flooded areas such as rice growing areas and human-made breeding sites in more arid savannahs, whereas the S form is more rain-dependant and is mostly observed during the wet period of the year [5]. Although the distribution of *An. arabiensis* is also influenced by eco-climatic variations, this species can now be found invading urban areas possibly as a consequence of adaptation to human activities/environments. The recent utilisation of artificial breeding sites by *An. arabiensis* has been documented in both East and West Africa [6-9]. In Burkina Faso previous studies showed that *An. arabiensis* was the third most prevalent malaria vector species after *An. gambiae s.s.* and *An. funestus* in western areas of the country [10-12] (Diabaté, unpublished) but was identified with similar frequency as the *An. gambiae* M form in central and eastern regions of the country (sudan-sahelian climate with moderate rainfall ranging from 600 to 1000 mm) [9]. In western regions of the country dominated by Sudan climatic conditions with relatively abundant rainfalls (1200 mm) the *An. gambiae* S form predominated, comprising up to 70% of the total *An. gambiae s.l.* population [5,13]. More recently in Bobo-Dioulasso city *An. arabiensis* was observed as a high proportion (up to 50%) of the total *An. gambiae s.l.* population and was identified at higher frequency than either *An. gambiae s.s.* forms [12]. These results were not in accordance with those of Robert *et al*. [14] and Diabaté *et al*. [15] who reported that the *An. gambiae* S form comprised more than 80% of the malaria vector population of Bobo-Dioulasso city. Taken together the sampling results in Bobo-Dioulasso city suggest that *An. arabiensis* populations may be gradually increasing in this area which was previously dominated by *An. gambiae s.s.* To investigate this possibility further the present study aims i) to describe the population dynamics of species within the *An. gambiae* complex occurring in Bobo-Dioulasso, ii) to update their insecticide resistance status and finally iii) to estimate the *Plasmodium* infection rates within these vector populations in two representative quarters of Bobo-Dioulasso with different environmental conditions.

## Methods

### Study areas

The study was carried out at two sites of Bobo-Dioulasso: Dioulassoba (11°10’42”N; 4°17’35”W), located in the center of the city and Kodéni (11°10’N; 4°15’W), located on the outskirts of the city. The two study sites represent two major environmental niches of the city characterised by the presence of vegetable cropping and the Houet stream. The Houet stream is a year-long flowing water source running through Dioulassoba whereas Kodeni is mostly characterised by temporary and semi-permanent wells created by small vegetable producers. The annual rainfall ranges from 1,000 to 1,200 mm.

### Mosquito collections

Specimens of *An. gambiae s.l.* were collected as adults and larvae in the two sites from May to December 2008. Larval stages were sampled from water bodies including, gutters and vegetable irrigation wells disseminated throughout the quarters and along the river Houet. Larval field collections were transferred to the laboratory for adult emergence and were then pooled. Adult *An. gambiae* mosquitoes were collected with other culicids by indoor spraying of aerosol insecticides early in the morning between 6,00-8,00 h a.m. in four houses during four consecutive days. They were then visually sorted from other anophelines according to morphological identification keys [16] and kept at −20°C for molecular and ELISA analysis.

### Estimation of the *Plasmodium* infection rates

The sporozoite infection rate of *An. gambiae s.l*. females collected indoors from May to December 2008 was estimated using the ELISA CSP technique [17].

### Insecticide susceptibility test

Insecticide susceptibility tests were performed on 2-3-day-old *An. gambiae s.l.* females using the WHO standard vertical tube protocol. Four insecticide-impregnated papers were used: 0.75% permethrin (cis:trans = 25:75), 0.05% deltamethrin, 0.1% bendiocarb and 4% DDT. The insecticide susceptibility status of wild-caught mosquitoes was compared with that of the “Kisumu” laboratory reference strain, which is fully susceptible to insecticides. Controls included “Kisumu” and wild-caught mosquitoes exposed to papers treated with solvent only. After 1 h exposure, mosquitoes were transferred into insecticide free tubes and maintained on sucrose solution. Final mortality was recorded 24 h after exposure. The threshold of susceptibility/resistance was fixed at 98% survivorship for the four insecticides according to the World Health Organisation (WHO) guidelines [18]. Dead and survivor mosquitoes were grouped separately and stored on silica gel at −20°C for subsequent PCR analysis.

### Molecular analysis

Genomic DNA was extracted from individual mosquitoes according to a slightly modified version of the procedure described by Collins and others [19]. After quantification of the extracted DNA, adults of *An. gambiae s.l.* including indoor spray catches tested in ELISA -CSP and samples of those tested in bioassay were processed by PCR for molecular identification of species and molecular form as described previously [20,21]. Bioassay survivors and those samples that died following exposure to each of the insecticides were further processed using additional PCR assays for the detection of *kdr* and *ace-1*^*R*^ alleles according to standard protocols [22,23].

### Statistical analysis

The frequency of each species, molecular form and sporozoite infection rates were compared between the two sites by chi-squared test. The frequency of the *kdr* mutation was calculated according to the formula *p* = 2AA + Aa/2n where AA was the number of homozygotes, Aa the number of heterozygotes and n the size of specimens analysed. As the sample size was sometimes too low to give reliable estimates of *kdr* and *ace-1*^*R*^ frequency for each species or molecular form, we pooled all specimens (resistant and susceptible to DDT, pyrethroids and bendiocarb) prior to allele frequency determination. The genotypic differentiation of *kdr* loci in *An. gambiae s.l.* populations was tested using the Fischer exact test implemented in GenePop (ver.3.4) software [24].

## Results

### Species composition, vector dynamics, and vectorial role of *An. arabiensis* in Kodeni and Dioulassoba

In total 1166 and 1119 mosquitoes were collected by indoor spraying of insecticide aerosol in Kodeni and Dioulassoba respectively from May to December 2008. *Anopheles* mosquitoes comprised a relatively low proportion of the total culicid collections in Kodeni and Dioulassoba (35.6% and 27% respectively). Of these, *An. gambiae s.l.*, was identified at the highest frequency *An. funestus* with *An. rufipes* representing less than 1%. The other culicids were composed exclusively of *Culex quinquefasciatus,* which reached 64.3% and 73% of the total collections at Kodeni and Dioulassoba respectively.

### Temporal population dynamics of *An. gambiaes.l*. and molecular forms at the two sites

**Figure 1:**
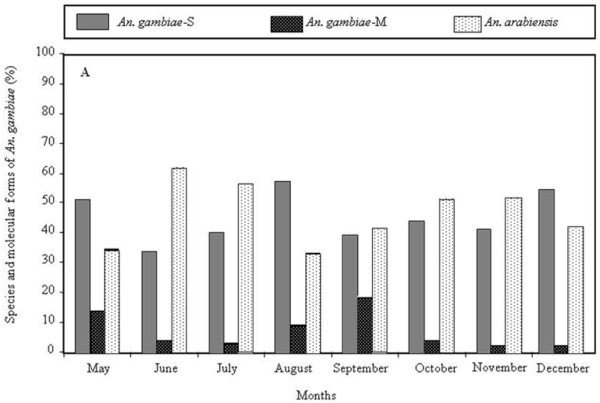
**Monthly population dynamics of*Anopheles gambiae s.l.*in Kodeni.**

**Figure 2:**
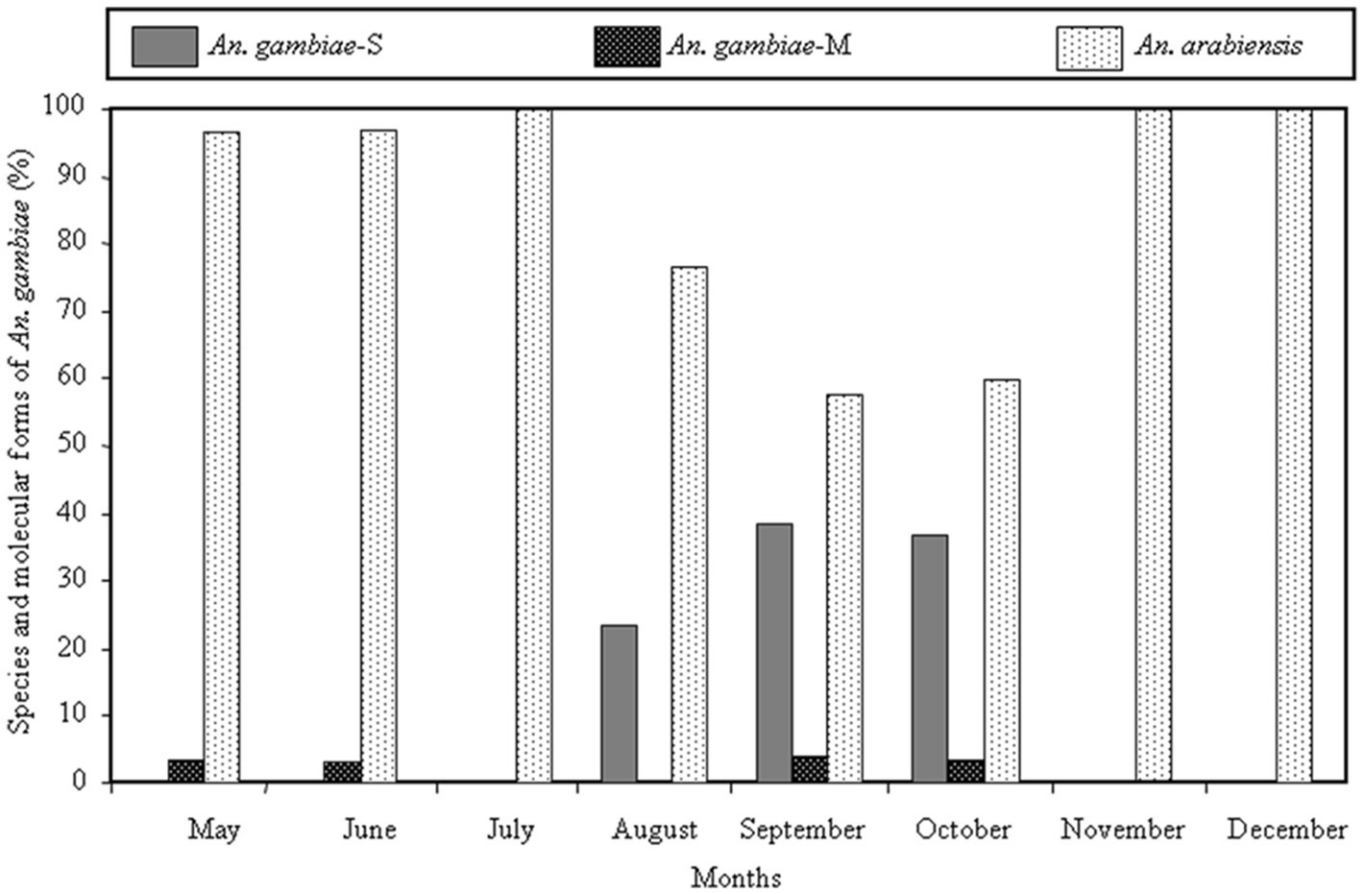
**Monthly population dynamics of*Anopheles gambiae s.l.*in Dioulassoba.**

In Kodeni the *An. gambiae* S form and *An. arabiensis* were the major species observed during the study period (Figure 1). Except for the first four months, they were collected in similar numbers. *An*. *gambiae* S form was found at higher frequency in May and August averaging 51 and 56% of collections *vs* 30% for *An*. *arabiensis*. In June and July *An*. *arabiensis* was more frequent peaking maximally in June. Throughout all months *An. gambiae* M form was found as a low proportion of total collections and was always less than 20% in any month (Figure 1). In contrast, in Dioulassoba regardless of the month *An. arabiensis* was always found at the highest frequency comprising up to 100% of collections in certain months (Figure 2). *An. gambiae* M form was only observed in collections during four months (May, June, August and September) where it comprised less than 5% of the monthly collection. The *An. gambiae* S form was collected in July, August and September, also at relatively low frequency (comprising less than 4% of the monthly collection) (Figure 2). Overall *An. arabiensis* was the major species sampled in Bobo-Dioulasso city during the study period.

### Sporozoite rates

Overall 658 *An. gambiae s.l*. females from the two study sites were analysed by ELISA CSP for infection with *Plasmodium falciparum* (Table 1). The mean infection rate (IR) was significantly higher in Kodeni (4.05%) than Dioulassoba (1.04%) (*χ*^2^ = 4.05; ddl = 1, *P* < 0.05). The infection rate was similar for *An. arabiensis* (6.2%) and *An. gambiae* S form (4.2%) in Kodeni with none of the, albeit smaller number of, *An. gambiae* M form mosquitoes infected. In Dioulassoba only a low percentage (1.4%) of *An. arabiensis* females were infected with none of the small number of *An. gambiae* M and S form mosquitoes tested infected. The highest percentage of infected females was found during October at the two sites (12/180 tested) although in Kodeni a few additional infected mosquitoes were observed in August and December.

**Table 1:** **Sporozoite rate calculated in*An. gambiae s.l.*collected from Kodeni and Dioulassoba by indoor insecticide aerosol spray (number in bracket is the percentage of infection)**

**Locality**	***An. gambiae S***		***An. gambiae M***		***An. arabiensis***		**Total tested**	**IR**
	**Nb tested**	**IR**	**Nb tested**	**IR**	**Nb tested**	**IR**		
Kodeni	191	8 [4.2]	66	0	113	7 [6.19]	370	15 [4.05]
Dioulassoba	52	0	24	0	212	3 [1.4]	288	3 [1.04]

### Susceptibility of *An. gambiae s.l.* populations to insecticides

Mortality rates were recorded 24 hours after the specimens were exposed to four insecticides: DDT, permethrin, deltamethrin and bendiocarb. The mortality rate in the control population was always 0%, therefore, Abbot’s correction was not necessary during data analysis. *An. gambiae s.l.* populations at the two sites were classified as resistant to DDT with mortality rates ranging from 18 to 58% (Table 2). In Kodeni the mortality rates of *An. gambiae s.l.* recorded in September 2008 were 85%, 94%, 86% respectively for permethrin, deltamethrin and bendiocarb corresponding to an intermediary status of resistance according to WHO guidelines [18]. The resistance was confirmed in November 2008 where low mortality rates ranging from 40% to 73% were recorded for the four insecticides. In Dioulassoba, *An. gambiae s.l.* populations exhibited an intermediary level of resistance to permethrin and deltamethrin in September and November and an intermediate level of resistance to bendiocarb in September (Table 2). PCR was used to assign *An. gambiae s.l.* that had survived or died in insecticide bioassays to species and molecular form (Table 3). This indicated that *An. arabiensis* populations at both sites were resistant to DDT (50% to 61% mortality), had an intermediate level of resistance to the pyrethroids, permethrin and deltamethrin (73% to 96% mortality) but were susceptible to bendiocarb. *An. gambiae* S form mosquitoes collected from both sites showed higher levels of resistance to all four insecticides than *An. arabiensis* (0-46% mortality to DDT, 51-73% mortality to the pyrethroids and 38-69% mortality to bendiocarb). *An. gambiae* M form mosquitoes collected from Kodeni were resistant to DDT but fully susceptible to bendiocarb, however, the number of M form mosquitoes collected was too low to assess their resistance status to the two pyrethroids in Kodeni or indeed to any insecticide at Dioulassoba (Tables 3,4 and 5).

**Table 2:** **Mortality rates of*An. gambiae s.l.*collected from Kodeni and Dioulassoba exposed to 4% DDT, 0.75% permethrin, 0.05% deltamethrin and 0.1% bendiocarb respectively**

**SITE**	**INSECTICIDE**							
	**4% DDT**		**0.75% Permethrin**		**0.05% Deltamethrin**		**0.1% Bendiocarb**	
	**N**	**Mortality%**	**N**	**Mortality%**	**N**	**Mortality%**	**N**	**Mortality%**
Kodeni								
September 08	100	43 (±2.06)	109	85.3 (±4.3)	101	94 (±0.5)	102	86.2 (±3.1)
November 08	105	40.1 (±0.57)	102	40.1 (±0.95)	98	73.4 (±1.4)	81	70.3 (±1)
Dioulassoba								
September 08	108	18.5 (±1.14)	95	68.4 (±6.1)	100	94 (±1.73)	114	85 (±0.95)
November 08	124	58 (±2.1)	107	88.7 (±2.5)	121	81.8 (±2.75)	97	100 (±3.05)

**Table 3:** **PCR identification of species and molecular form of*An. gambiae s.l.*mosquitoes collected from Kodeni and Dioulassoba and tested in insecticide exposure assays**

**Sites**	***An. gambiae s.l***.**N tested in PCR**	***An. gambiae*S**	***An. gambiae*M**	***An. arabiensis***
**Kodeni**				
**4% DDT**				
September 08	52	**8***(7)* [46%]	**15***(9)* [38%]	**5***(8)* [61%]
November 08	30	**20***(0)* [0%]	**1***(0)*	**1***(8)*
**0.75% Permethrin**				
September 08	106	**30***(32)* [51%]	**6***(1)*	**6***(31)* [83%]
November 08	42	**11***(10)* [48%]	**0***(0)*	**2***(19)* [90%]
**0.05% Deltamethrin**				
September 08	101	**15***(30)* [67%]	**0***(1)*	**2***(53)* [96%]
November 08	41	**4***(11)* [73%]	**3***(0)*	**4***(19)* [83%]
**0.1% Bendiocarb**				
September 08	75	**16***(10)* [38%]	**0***(14)* [100%]	**0***(35)* [100%]
November 08	32	**4***(6)* [60%]	**0***(0)*	**0***(22)* [100%]
**Dioulassoba**				
**4% DDT**				
September 08	61	**13***(0)* [0%]	**0***(1)*	**19***(28)* [60%]
November 08	40	0*(0)*	0*(0)*	20*(20)* [50%]
**0.75% Permethrin**				
September 08	77	**16***(22)* [58%]	**0***(2)*	**5***(32)* [86%]
November 08	30	0	0	8*(22)* [73%]
**0.05% Deltamethrin**				
September 08	63	**13***(18)* [58%]	**2***(4)*	**1***(25)* [96%]
November 08	36	**0***(0)*	**0***(0)*	**6***(30)* [83%]
**0.1% Bendiocarb**				
September 08	67	**5***(11)* [69%]	**0***(0)*	**1***(50)* [98%]
November 08	34	**0***(0)*	**0***(0)*	**0***(34)* [100%]

N = number of samples tested in PCR; numbers in bold represent the number of survivors; numbers in *italics* represent the number of specimens that died; the figure in square brackets represents the percentage mortality for each species or molecular form when n ≥ 10.

**Table 4:** **Frequency of the*kdr*L1014F mutation in*An. gambiae s.l.*from Kodeni and Dioulassoba that were tested against 4% DDT and pyrethroids (0.75% permethrin and 0.05% deltamethrin)**

**Locality**	**Species**	**N**	**SS**	**RS**	**RR**	***F(kdr)***	HW (P-value)*
Kodeni	*An. arabiensis*	127	127	0	0	0	-
	*An. gambiae* -M	22	8	8	6	0.45	-
	*An. gambiae* -S	145	3	16	126	0.92	0.013
Dioulassoba	*An. arabiensis*	208	208	0	0	0	-
	*An. gambiae* -M	8	4	0	4	0.5	0.03
	*An. gambiae* -S	76	5	1	70	0.92	0.001

F(*kdr*) values = frequencies of the *kdr* mutation. HW = Hardy-Weinberg test.

*The exact probability for rejecting Hardy–Weinberg equilibrium.

The genotype of each mosquito specimen tested is also shown.

**Table 5:** **Frequency of the*ace-1***^***R***^**allele in*An. gambiae s.l.*from Kodeni and Dioulassoba tested against 0.1% bendiocarb. The genotype of each mosquito specimen is also shown**

**Locality**	**Species**	**N**	**SS**	**RS**	**RR**	***F(ace-1***^***R***^*)*	HW (P-value)*
Kodéni	*An. arabiensis*	57	57	0	0	0	-
	*An. gambiae* -M	14	14	0	0	0	-
	*An. gambiae* -S	39	16	22	1	0.3	0.999
Dioulassoba	*An. arabiensis*	70	70	0	0	0	-
	*An. gambiae* -M	0	0	0	0	0	-
	*An. gambiae* -S	17	8	8	1	0.29	0.843

F(*ace-1*^*R*^) values = frequencies of the *ace-1*^*R*^ mutation. HW = Hardy-Weinberg test.

*The exact probability for rejecting Hardy–Weinberg equilibrium.

### Frequency of the *kdr* mutation (L1014F) in *An. gambiae s.l.* population

The frequency of the *kdr* mutation was not directly compared to resistance (as determined by insecticide exposure assays), in this study as to maximize the sample size for each species specimens that had survived or died upon exposure to pyrethroids and DDT were pooled. In total 586 specimens including resistant and susceptible mosquitoes exposed to DDT, permethrin and deltamethrin were tested. The *kdr* mutation (L1014F) was found at high frequency (0.92) in *An. gambiae s.s.* S form specimens from both sites (Table 2). The *kdr* mutation was observed at lower frequency (~0.5) in M-form specimens and was found at similar frequency in the populations at both sites, however, the sample size for this species was relatively low. The *kdr* mutation was not identified in *An. arabiensis* from either site. The S-form populations from both sites showed a higher than expected number of individuals homozygous for the *kdr* mutation rejecting Hardy–Weinberg equilibrium at these sites (*P* = 0.013 &*P* = 0.001 respectively in Kodeni and Dioulassoba).

### Frequency of the *ace-1*^*R*^ mutation in *An. gambiae s.l.*

Overall 105 specimens of *An. gambiae s.l.* exposed to bendiocarb including 18 resistant and 87 susceptible mosquitoes were analysed by PCR in Kodeni for the detection of the *ace-1*^*R*^ mutation (G119). The mutation was detected in 17 of the 18 resistant specimens (16 heterozygous 1 homozygous, a frequency of 0.53). All of the specimens carrying the *ace-1*^*R*^ mutation were of the *An. gambiae s.s.* S form.

In Dioulassoba, of 87 specimens of *An. gambiae s.l.* the *ace-1*^*R*^ mutation was only identified within *An. gambiae s.s.* S form individuals at a frequency of 0.29 with 1 homozygous and 8 heterozygous individuals. All individuals that survived bendiocarb exposure carried both the *kdr* and *ace-1*^R^ mutations and were homozygous for the *kdr* mutation. No *An. arabiensis* was identified carrying the *ace-1*^*R*^ mutation at either site.

Sample numbers were sufficient to compare *ace-1*^*R*^ gene frequencies with Hardy-Weinberg expectations in populations collected from the two sites (when n = 14 or greater). The observed genotypic frequencies were not significantly different from Hardy-Weinberg expectations at the 95% confidence level in populations from either site (Table 3).

## Discussion

The *An. gambiae* complex is composed of at least seven morphologically indistinguishable species [1,25] throughout sub-Saharan Africa including neighbouring islands. Among them only *An. gambiae s.s*. and *An. arabiensis* are found in Burkina Faso. These species are sympatric in the major parts of the country but the relative frequency of the two species varies in rural and urban areas. Previously, Coluzzi and others [25] reported penetration of *An. arabiensis* into towns and cities of the rainy forest zone in southern Nigeria. Kristan and others [26] reported a similar trend among samples *of An. gambiae s.l.*, in the urban localities of Aiyetoro and Lantoko of Nigeria where the majority of the vector population was identified as *An. arabiensis*. Although Lemasson and others [27], showed that *An. arabiensis* had a lower vectorial capacity than *An. gambiae s.s.* in Senegal, these results imply an extension/adaptation of this species into/to urban areas. Studies in more locations are needed to further confirm and understand what may be driving the expansion of *An. arabiensis* in West African cities. *An. arabiensis* is the most widespread species among the members of the *An. gambiae* complex and is the most adaptive in respect to feeding and resting choices [28,29]. In 1986, Robert and others [14] studying malaria transmission in Bobo-Dioulasso city including Dioulassoba (the same site as the present study) identified only 3% of the mosquito malaria vector population as *An. arabiensis*. In 1999, Chandre and others [30] failed to identify any *An. arabiensis* in the same area. In 2002, Diabaté and others recorded that the malaria vector population in Dioulassoba was composed of 8.3% *An. arabiensis*[15]. Our results show the advanced infiltration of *An. arabiensis* into this district where its proportion of the total vector composition now reaches 90% whatever the sampling period. The same situation has also been observed in Kodeni, a peripheral district of the city (>50% *An. arabiensis vs* 40% *An. gambiae s.s.*). According to a recent study, *An*. *arabiensis* was also reported as the dominant vector in the savannah around Bobo-Dioulasso city suggesting that this infiltration now extends beyond Bobo-Dioulasso [31]. Indeed, while the frequency and distribution of *An. arabiensis* appears to be growing, the role of secondary vectors such as *Anopheles nili* which had previously played a local but important role in malaria transmission in rural areas surrounding Bobo-Dioulasso seems to be greatly reduced [11]. The pattern of *An. arabiensis* expansion in this region could be explained by global ecological changes (such as climate change) or local human activities favouring the colonisation of this species, however, further investigation is needed to examine these two possibilities further. The trend identified in our study and those of others appears to be mirrored in the capital of Burkina Faso, Ouagadougou, where *An. gambiae s.s.* was formerly reported as the major vector species [32,33]. However, recently *An. arabiensis* has been described as the predominant vector species in this city (55% *An. arabiensis vs* 45% for the *An. gambiae* M form) [9]. In *An. gambiae* M and S forms adaptation to different ecological niches is associated with specific chromosomal inversions that appear to confer on the M form traits that allow exploitation of flooded and arid areas and make the S form significantly rain dependant [4,34]. However, more investigation is needed to understand the genetic basis underlying the adaptation of *An*. *arabiensis* either to the humid meridian savannah or to polluted sites in urban areas as seems to be the case in our study.

Considering the changing pattern of vector bionomics in Bobo-Dioulasso over the last decade or so it might be assumed that the malaria transmission potential has altered. In 1986, a low annual entomological inoculation rate (EIR) of 0.19 infected bites per year (i/b/y) in Dioulassoba and 4.6 i/b/y in peripheral districts had been reported [14]. In 2003 Diabaté (unpublished data) recorded higher inoculation rates reaching 57 i/b/y in Dioulassoba and 63 i/b/y in peripheral districts. In the current study our sampling technique did not allow the EIR to be estimated with any accuracy. However, taking into account mosquito infection rates alone it is possible that the transmission intensity may not have changed greatly from that formerly reported in 2003 by Diabaté (unpublished data).

To test the susceptibility of these urban vector populations to insecticides we exposed them to the most commonly used insecticides for public heath purposes and also to DDT. The frequency of two common resistance mechanisms in these populations, *kdr* and *ace-1*^*R*^, that confer resistance to pyrethroids/DDT and organophosphates/carbamates respectively was also examined. Taking the *An. gambiae s.l.* population as a whole, resistance was observed to pyrethroids, bendiocarb and most significantly to DDT at both sites. The *An. gambiae* S form showed the highest levels of resistance to all four insecticides and this was consistent with the high frequency of *kdr*/*ace-1*^*R*^ observed in this molecular form, the former of which appears to be approaching fixation. Because of the relative rarity of the *An. gambiae* M form it was not possible to assess its resistance status to all insecticides, however, it showed clear resistance to DDT but was fully susceptible to bendiocarb, consistent with a frequency of ~0.5 for *kdr* and an absence of *ace-1*^*R*^. *An. arabiensis* populations also showed resistance to DDT, modest levels of resistance to pyrethroids, and were fully susceptible to bendiocarb. In contrast to the *An. gambiae* S form the L1014F *kdr* mutation was not found in *An. arabiensis* suggesting that other mechanism(s) underlie resistance to DDT/pyrethroids. It would therefore be interesting in future to investigate the role of detoxifying enzymes such as esterases, cytochrome P450s and glutathione-s-transferases in resistance. No *An. arabiensis* was found to carry the *ace-1*^*R*^ mutation correlating with the results of bioassays showing that *An. arabiensis* remains susceptible to carbamate compounds.

The resistance level observed in *An. gambiae s.s.* at the two sites may be partly explained by the use of insecticides for crop protection in Kodeni where farmers apply large amounts of insecticides for vegetable production and the domestic use of insecticides such as aerosol sprays or coils in Dioulassoba. In 1993 in Bouaké, Côte d'Ivoire, the presence of permethrin resistance was attributed to widespread use of pyrethroids in households [35].

In this study *An. gambiae s.s.* mosquitoes carrying both *kdr* and *ace-1*^*R*^ mutations were identified in the field. The presence of both these resistance mechanisms in *An. gambiae s.s.* from the West of Burkina Faso has been reported previously and would be expected to provide a level of protection to pyrethroids, carbamates and organophosphates [36]. Presently, pyrethroid treated bednets alone or combined with indoor residual spraying remain the primary mechanism to control malaria vectors in these regions. An essential component of effective vector management strategies is the monitoring of vector populations for resistance to the insecticides used for control and the frequency and distribution of mechanism(s) underlying resistance. The data our study provides is therefore useful contemporary information for vector control programmes in Bobo-Dioulasso city.

## Conclusion

This study demonstrates that the population dynamics of malaria vectors in one area can change with time, possibly in response to climate change or human activities. However, changes in these populations may not necessarily significantly impact malaria transmission. Our study confirms that *An*. *arabiensis,* formerly comprising a low percentage of the vector population in the south of the country, is replacing *An. gambiae s.s.* as the major vector species in Bobo-Dioulasso city and the savannah villages surrounding the city. This change in vector composition occurring in many parts of Africa is not new but is important as it has significant potential impacts for vector control strategies [37,38]. For example, *An. arabiensis* has been shown to be more exophilic than *An. gambiae s.s.* and this can reduce the effectiveness of vector control interventions that specifically target endophilic and anthropophilic species.

## Competing interest

The authors declare no competing interest.

## Authors’ contribution

RKD participated in the study design, supervised the field study, analysed the data and wrote the paper. MN participated in conducting bioassays performed in the laboratory and related data analysis. LBY and AO participated in sample collection and analysis in the laboratory. HKT participated in the molecular analysis in the lab. SPS participated in the statistical analysis. FS, FC, LCG and TB and CB participated in the drafting and the revision to the paper. AD participated in the manuscript drafting. All authors approved the final version of the manuscript.
